# Deletion of the Voltage-Gated Calcium Channel Gene, Ca_V_1.3, Reduces Purkinje Cell Dendritic Complexity Without Altering Cerebellar-Mediated Eyeblink Conditioning

**DOI:** 10.1007/s12311-025-01914-y

**Published:** 2025-10-11

**Authors:** Annette Klomp, Martha Pace, Jacqueline Mehr, Maria Fernanda Hermosillo Arrieta, Cessily Hayes, Anthony Fleck, Shane Heiney, Aislinn Williams

**Affiliations:** 1 Department of Psychiatry, University of Iowa, Iowa City, IA USA; 2 Graduate Program in Neuroscience, University of Iowa, Iowa City, IA, USA; 3 Department of Biology, Upper Iowa University, Fayette, IA USA; 4 Department of Psychiatry, Brain Health Institute, Robert Wood Johnson Medical School, Rutgers University, Piscataway, NJ USA; 5 Iowa: Discovering Research Experiences and Mentorship, University of Iowa, Iowa City, IA USA; 6 Iowa Neuroscience Institute, Iowa City, IA USA

**Keywords:** Purkinje cells, Eyelid conditioning, Association learning, Cerebellar cortex, Cacna1d, Ca_V_1.3

## Abstract

Genetic variation in *CACNA1D*, the gene that encodes the pore-forming subunit of the L-type calcium channel Ca_V_1.3, has been associated with increased risk for neuropsychiatric disorders that display abnormalities in cerebellar structures. We sought to clarify if deletion of Ca_V_1.3 in mice would induce abnormalities in cerebellar cortex cytoarchitecture or synapse morphology. Since Ca_V_1.3 is highly expressed in cerebellar molecular layer interneurons (MLIs) and L-type channels appear to regulate GABA release from MLIs, we hypothesized that loss of Ca_V_1.3 would alter GABAergic synapses between MLIs and Purkinje cells (PCs) without altering MLI density or PC structure. As expected, we did not observe changes in the density of MLIs or PCs. Surprisingly, Ca_V_1.3 KO mice do have decreased complexity of PC dendritic arbors without differences in the number or structure of GABAergic synapses onto PCs. Loss of Ca_V_1.3 was not associated with impaired acquisition of delay eyeblink conditioning. Therefore, our data suggest that Ca_V_1.3 expression is important for PC structure but does not affect other measures of cerebellar cortex morphology or cerebellar function as assessed by delay eyeblink conditioning.

## Introduction

Genetic variation in *CACNA1D*, the gene that encodes the pore-forming subunit of the L-type calcium channel Ca_V_1.3, has been associated with increased risk for autism spectrum disorder [23, 55–58], bipolar I disorder [2, 61], epilepsy [55, 57], schizophrenia [14, 50], hyperaldosteronism [22, 48, 54–57, 64, 70], and deafness and bradycardia [5] suggesting that Ca_V_1.3 plays an important role in a wide variety of systems. Schizophrenia, autism spectrum disorder, and bipolar disorder subjects display abnormalities in cerebellar volume, gene expression, connectivity patterns of cerebellar circuits, and cerebellar-dependent motor and cognitive behaviors [3, 4, 6, 8, 13, 27, 37, 43, 44, 51, 66, 69]. However, the cellular mechanisms that drive changes in the cerebella of patients with these neuropsychiatric disorders are still unknown. Given the genetic links between L-type calcium channels and neuropsychiatric disorders, a better understanding of the role of L-type channels in cerebellar microcircuit structure may be broadly applicable to our understanding of neuropsychiatric disorders.

One possible role for L-type calcium channels in the cerebellum is in modulating the development or function of the cerebellar cortex microcircuit. In the cerebellar cortex microcircuit, PCs receive excitatory input from climbing fibers from the inferior olive and the parallel fibers of granule cells, as well as inhibitory signals from MLIs [19, 20, 29], and PCs then send inhibitory signals to the deep cerebellar nuclei [16, 75]. This cerebellar circuitry initiates and coordinates movement, various cognitive processes including aspects of learning and memory, and emotional states [15, 24, 53, 63, 71, 73]. MLIs consist of two subsets of neurons: basket cells (BCs) which generally reside in the basal one-third of the molecular layer, and stellate cells (SCs) which typically reside in the apical two-thirds of the molecular layer [49]. BC axons wrap around and synapse on the initial segment of the PC axon via a structure termed a pinceau [11], whereas SCs are generally located more superficially in the molecular layer and synapse onto the dendrites of PCs [20].

Several cerebellar cell types, such as PCs, Golgi cells, and MLIs, express *Cacna1d* mRNA according to mouse single-nucleus transcriptomic datasets [40, 62] and immunohistochemical and proteomic data [32, 74]. L-type channel activity has been detected in developing immature PCs but is less apparent in mature PCs [26, 72], suggesting that Ca_V_1.3 in PCs may be important primarily in development. L-type currents have been detected in MLIs and are thought to modulate GABA release from MLIs to PCs [60], suggesting a potential role for Ca_V_1.3 in mature MLI function.

We explored whether ubiquitous germline deletion of Ca_V_1.3 results in abnormal morphological features in PCs and MLIs, and whether this alters cerebellum-dependent learning using the delay eyeblink conditioning paradigm. Given the high expression and electrophysiological role of Ca_V_1.3 in cerebellar MLIs, we predicted that loss of Ca_V_1.3 would alter MLIs and the GABAergic synapse between MLIs and PCs. However, we have instead found that Ca_V_1.3 deletion alters PC morphology without appearing to affect the synaptic structure or fate specification of MLIs, and without impacting acquisition of cerebellum-dependent delay eyeblink conditioning.

## Methods

### Mice

The generation of Ca_V_1.3 knockout (KO) mice (Cacna1d^tm1Jst^) has been described previously [42, 59]. Breeding pairs of Ca_V_1.3^+/−^ mice were maintained on a C57BL/6NTac background by crossing Ca_V_1.3^+/−^ offspring with C57BL/6NTac wild-type (WT) mice purchased from Taconic Biosciences (Rensselaer, NY). To generate experimental animals, Ca_V_1.3^+/−^ males were bred to Ca_V_1.3^+/−^ females to obtain male and female Ca_V_1.3 WT and KO littermates. All mice were adults at the time of use. Sample sizes and ages are indicated in each figure legend. All experiments were carried out in a manner to minimize pain and discomfort. All experiments were conducted according to the National Institute of Health guidelines for animal care and were approved by the Institutional Animal Care and Use Committee at University of Iowa.

### Histology

Ca_V_1.3 KO and wild type littermates 13–30 weeks old (*n* = 6–8 per genotype) were anesthetized with 17.5 mg/ml Ketamine/2.5 mg/ml Xylazine at a dose of 0.1 ml per 20 g and perfused with 4% paraformaldehyde in 0.1 M phosphate buffer (PB). Whole brains were dissected and immersed in 30% sucrose for 72 h. Brains were rinsed in PB and frozen in optimal cutting temperature compound. Brain tissue was serially sectioned on a cryostat into 20-µm-thick sagittal sections, mounted on slides, and stored at −20 °C until use. All histology was performed in cerebellar vermis.

### Immunostaining

Blocking buffer was made from normal donkey serum (5%) and Triton-X (0.1%) in PB. Primary antibodies used included: rabbit anti-Calbindin D28K (ThermoFisher, Cat: 711 − 443) 1:250, mouse anti-parvalbumin (Swant, Cat: PV 235) 1:250, rabbit anti-HCN1 (Synaptic Systems, Cat: 338-003) 1:50, mouse anti-GAD6 (DSHB, Cat: AB_528264) 1:250, and mouse anti-Aldolase C 4A9 (Novus Bio, Cat: NBP2-25145) 1:500. All primary antibodies were incubated on sections overnight at 4 °C. Secondary antibodies used included donkey anti-rabbit 488 (Jackson, Cat: 711-545-152) 1:500 and donkey anti-mouse 594 (Jackson, Cat: 715-585-151) 1:500. Secondaries for Aldolase C labeling were incubated overnight at 4 °C and all other secondaries were incubated at RT for 2–4 h. Sections were incubated with DAPI Solution (Thermo Scientific) 1:1000 for 1 min and coverslips were mounted with Prolong Diamond Antifade Mountant (Invitrogen).

### Golgi Staining

Ca_V_1.3 KO and littermate wild type mice (*n* = 3 per genotype, all 15 weeks old) were anesthetized with isoflurane. Whole brains were dissected and stained with the FD Rapid GolgiStain Kit (FD NeuroTechnologies). Brains were immersed in impregnation solution A/B for 2 weeks then immersed in solution C for 5 days before sectioning at 100 μm and staining sections with staining solution D/E per manufacturer protocol. Sections were dehydrated with ethanol and coverslips were mounted with Prolong Diamond Antifade Mountant (Invitrogen). For analysis, Purkinje cells were sampled throughout the vermis.

### Microscopy

Sections were imaged either at 20x on an Olympus IX83 fluorescence microscope and stitched together using Olympus CellSens Dimension 2.3 software or at 40x in Z-stacks on a Leica SPE Confocal Microscope. Analyses were performed with ImageJ or Imaris 9.9.1 by raters blinded to genotype. For measurements of layer thickness and cell density, the image scale was calibrated on ImageJ by measuring the length of the scale bar (known distance) to convert pixels to microns. For layer thickness measurements, a perpendicular line was drawn to the width of the granule or molecular layer in each lobule and the line was measured. This process was repeated three times throughout each lobule in each histological section, then averaged across the lobule for each mouse. PCs were quantified in serial histological sections at least 100 μm apart. PCs were counted by drawing a straight line across the PC monolayer and counting the PCs intersecting the line. This number was then divided by the length of the line to determine PC density in each lobule. Filament tracing for Sholl analysis on Golgi-Cox stained sections was done with the Imaris Filaments tool [18]. For dendrite tracing, Imaris settings were set to largest diameter of 10 μm and thinnest diameter of 1 μm. Dendritic spines were quantified using calbindin labeling of Purkinje cells. To detect dendritic spines, Imaris settings were set to thinnest diameter spine head of 0.3 μm and max spine length of 4 μm. Spine classifications were made using the standard default settings in Imaris. For quantification of GABAergic synapses on calbindin-positive PC dendrites, Imaris settings were set to thinnest diameter spine head of 0.3 μm and max spine length of 1 μm. For quantification of pinceaux size, we used the method described in [77] with some modifications. Briefly, images of cerebellar cortex sections double labeled with HCN1 and Aldolase C were loaded into ImageJ/Fiji, and the images were thresholded such that the pinceaux appeared filled. We used the drawing tool in ImageJ to outline the HCN1-positive pinceaux at the axon initial segment of the PC axon, then used ImageJ to measure the cross-sectional area.

### Eyeblink Conditioning

Delay eyeblink conditioning was performed as previously described [30, 31]. Briefly, mice were implanted with a titanium headplate and habituated to head fixation on a treadmill for 2 days. During habituation, no stimuli were presented. Training was performed for 9 days and consisted of 100 trials/day, with 90 CS-US paired trials and 10 conditioned stimulus (CS)-alone trials pseudorandomly interleaved. Mice were free to walk on the treadmill during the entire session. The CS was an LED, and the unconditioned stimulus (US) was a puff of air (20 PSI source pressure) of 20–30 ms duration directed at the cornea via a 23-gauge needle placed 3 mm from the mouse’s eye. These pulse durations resulted in 6–8 PSI puff intensities measured at the end of the needle. Interstimulus interval was set at 250 ms. CS-alone trials were included to assess conditioned responses (CRs) uncontaminated by the US or unconditioned response (UR). Trials were separated by a variable inter-trial interval (ITI) that averaged 15–25 s.

### Statistical Analysis

Data were graphed and analyzed using GraphPad Prism 9.0 (GraphPad Software, San Diego, CA) and R (R 4.1.1, emmeans 1.7.0, lme4 1.1.27.1, lmerTest 3.1-3, effectsize 0.5), except for eyeblink conditioning figures which were generated in Adobe Illustrator from MATLAB plots. Data are graphically represented as mean ± standard error of the mean (SEM) for each group. Data were analyzed using the statistical test noted in results (linear mixed model, two-way repeated measures ANOVA, one-way ANOVA, or Student’s *t*-tests with appropriate follow-up testing). Behavioral data from Ca_V_1.3 KO mice do not show strong sex by genotype interaction effects [42, 46], so we have combined sexes for histological experiments. Eyeblink conditioning data were analyzed and reported in the results including sex as a variable, but since no sex differences were observed, results are graphed with males and females combined. Results were considered significant when *p* < 0.05 (denoted in all graphs as follows: *p < 0.05; **p < 0.01).

## Results

### Loss of Ca_V_1.3 does not Alter Cerebellar Cortex Thickness

**Fig. 1:**
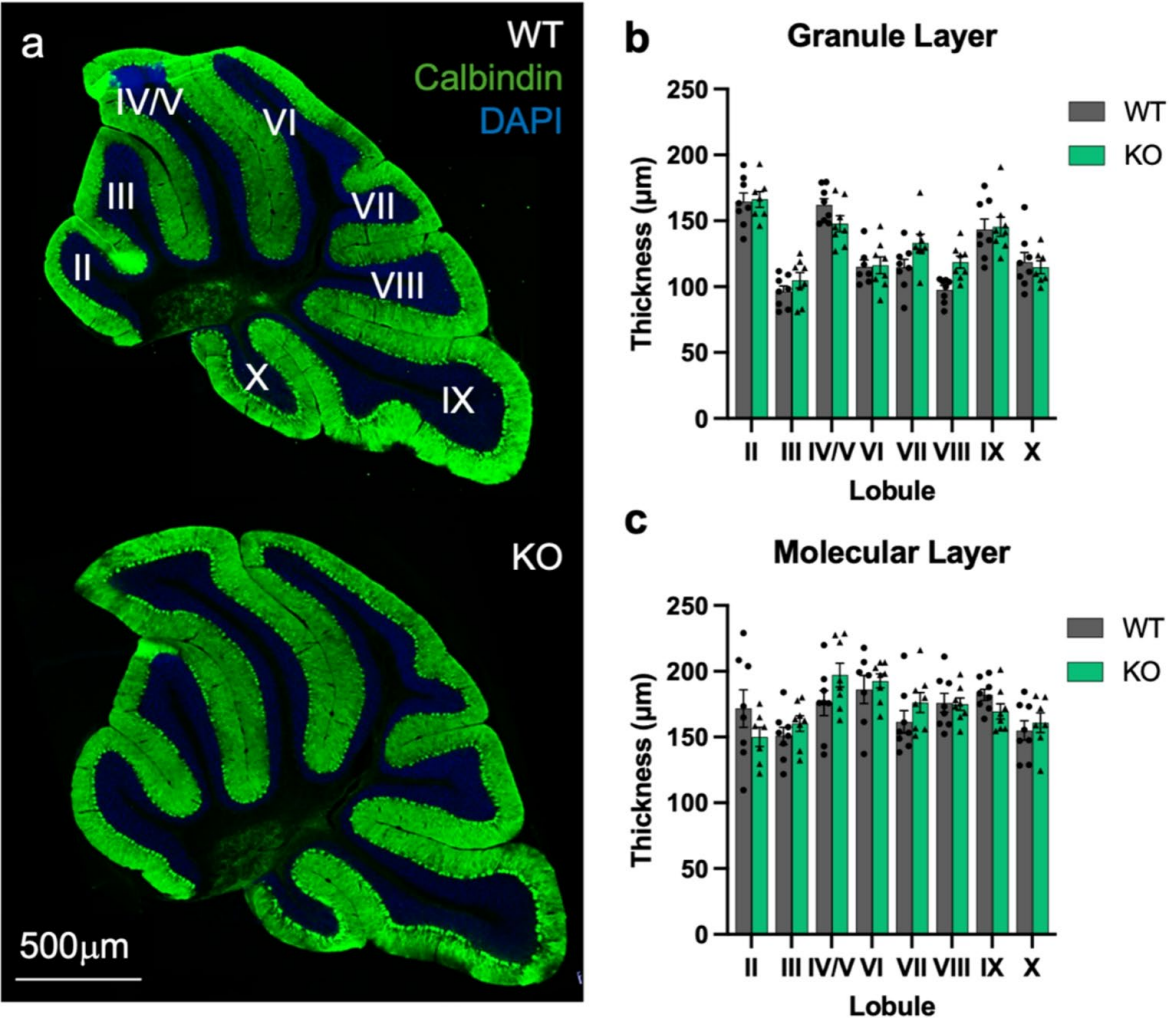
Cerebellar cortex layer thickness varies by lobule. (**a**) Immunofluorescence labeling of calbindin (green) and DAPI (blue) in sagittal sections of cerebellar vermis in WT (top) littermates and Ca_V_1.3 KO (bottom) littermates. Cerebellar lobule numerals are labeled in white. Although cortical layer thicknesses vary across lobules, deletion of Ca_V_1.3 does not alter granule layer (**b**) or molecular layer (**c**) thickness. Data are expressed as mean ± s.e.m. *N* = 7–8/genotype, aged 21–33 weeks

Given the high expression of *Cacna1d* in cerebellar MLIs [40, 62] and known roles for L-type channels in neuronal structure in hippocampal neurons [38, 67, 68], we hypothesized that Ca_V_1.3 is important for normal cerebellar morphology. We first sought to determine whether Ca_V_1.3 KO mice display abnormal cerebellar cortex layer thickness (Fig. 1a). We observed no Ca_V_1.3-dependent differences in granular layer thickness in any lobule (*n* = 8/genotype, 2-way ANOVA, main genotype effect, F_1,109_=2.15, *p* = 0.15, genotype x lobule interaction effect, F_7,109_=1.89, *p* = 0.08) (Fig. 1b) or molecular layer thickness in any lobule (*n* = 8/genotype, 2-way ANOVA, main genotype effect, F_1,109_=0.50, *p* = 0.48, genotype x lobule interaction effect, F_7,109_=1.56, *p* = 0.15) (Fig. 1c). We did observe granular and molecular layer thickness differences between lobules that were independent of genotype (granular layer thickness; main effect lobule, F_7,109_=30.51, *p* < 0.01; molecular layer thickness; main effect lobule, F_7,109_=5.09, *p* < 0.01) (Fig. 1b-c). Overall, deletion of Ca_V_1.3 does not appear to alter cerebellar granular or molecular layer thickness.

### Loss of Ca_V_1.3 does not Alter PC or MLI Density

**Fig. 2:**
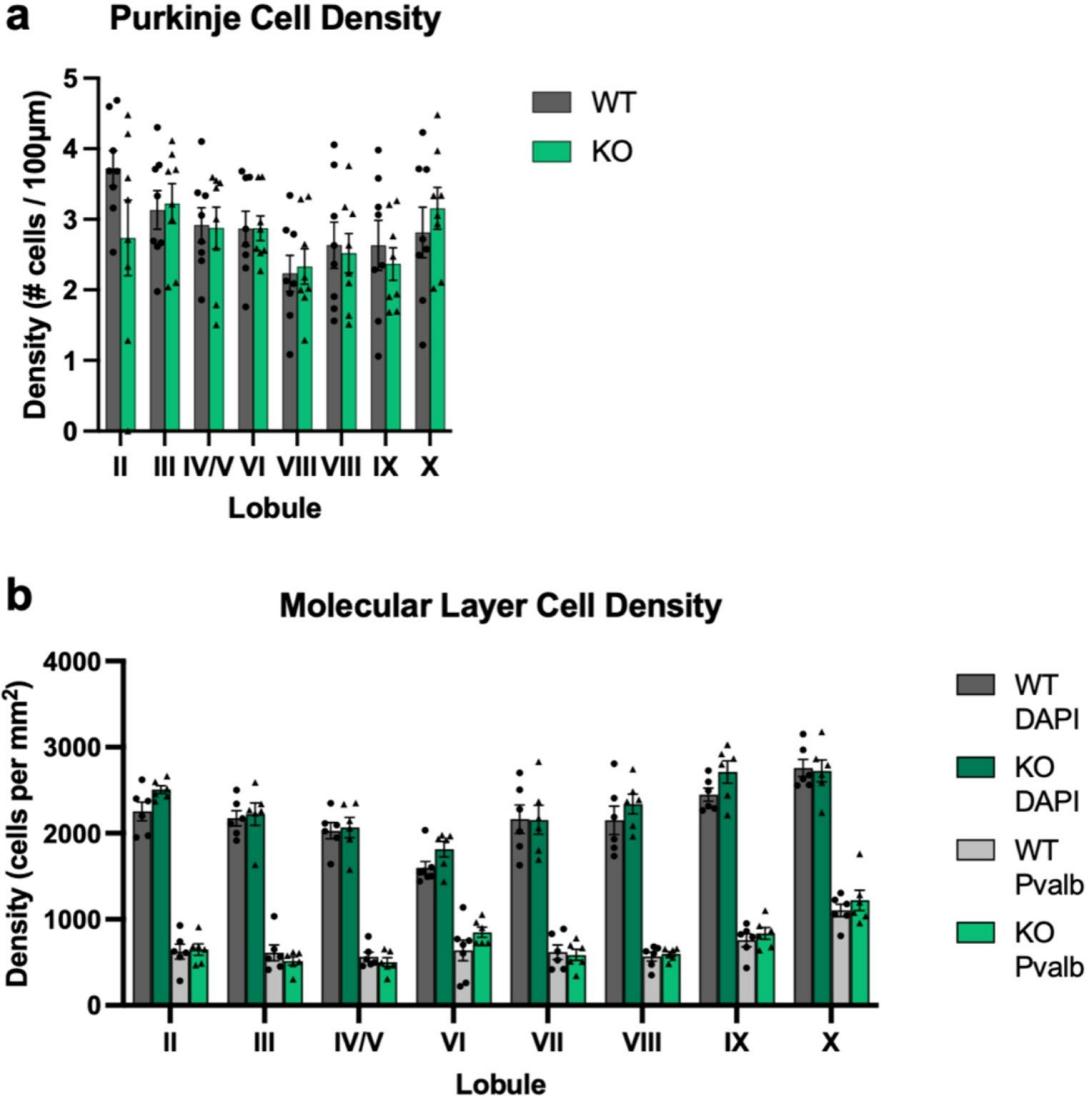
MLI density varies by lobule. Deletion of Ca_V_1.3 does not alter PC density (**a**) or MLI density (**b**), although both PC and MLI densities statistically vary as a function of lobule. Data are expressed as mean ± s.e.m. *N* = 7–8/genotype, aged 21–33 weeks

We next sought to determine whether Ca_V_1.3 KO mice display abnormal density of PCs or MLIs. We observed no genotype-dependent differences in density of PCs although we did observe a main effect of lobule (*n* = 8/genotype, 2-way ANOVA, main genotype effect, F_1,112_=0.52, *p* = 0.47, main lobule effect, F_7,112_=2.46, *p* = 0.02, genotype x lobule interaction effect, F_7,112_=0.86, *p* = 0.54) (Fig. 2a). We also observed no genotype-dependent differences in density of MLIs (*n* = 6/genotype, DAPI, Linear Mixed Effects Model, main genotype effect, F_1,11_=2.13, *p* = 0.17, genotype x lobule interaction effect, F_7,71_=0.65, *p* = 0.71; PV, Linear Mixed Effects Model, main genotype effect, F_1,7_=0.01, *p* = 0.92, genotype x lobule interaction effect, F_7,66_=1.48, *p* = 0.19) (Fig. 2b) in any lobule of the vermis. As with granule and molecular layer thickness, we did observe lobule-dependent differences in MLI density that were independent of genotype (DAPI; main lobule effect, F_7,71_=18.34, *p* < 0.01; PV; main lobule effect, F_7,66_=20.74, *p* < 0.01) (Fig. 2b).

### Loss of Ca_V_1.3 Reduces Complexity of PC Dendrites

**Fig. 3:**
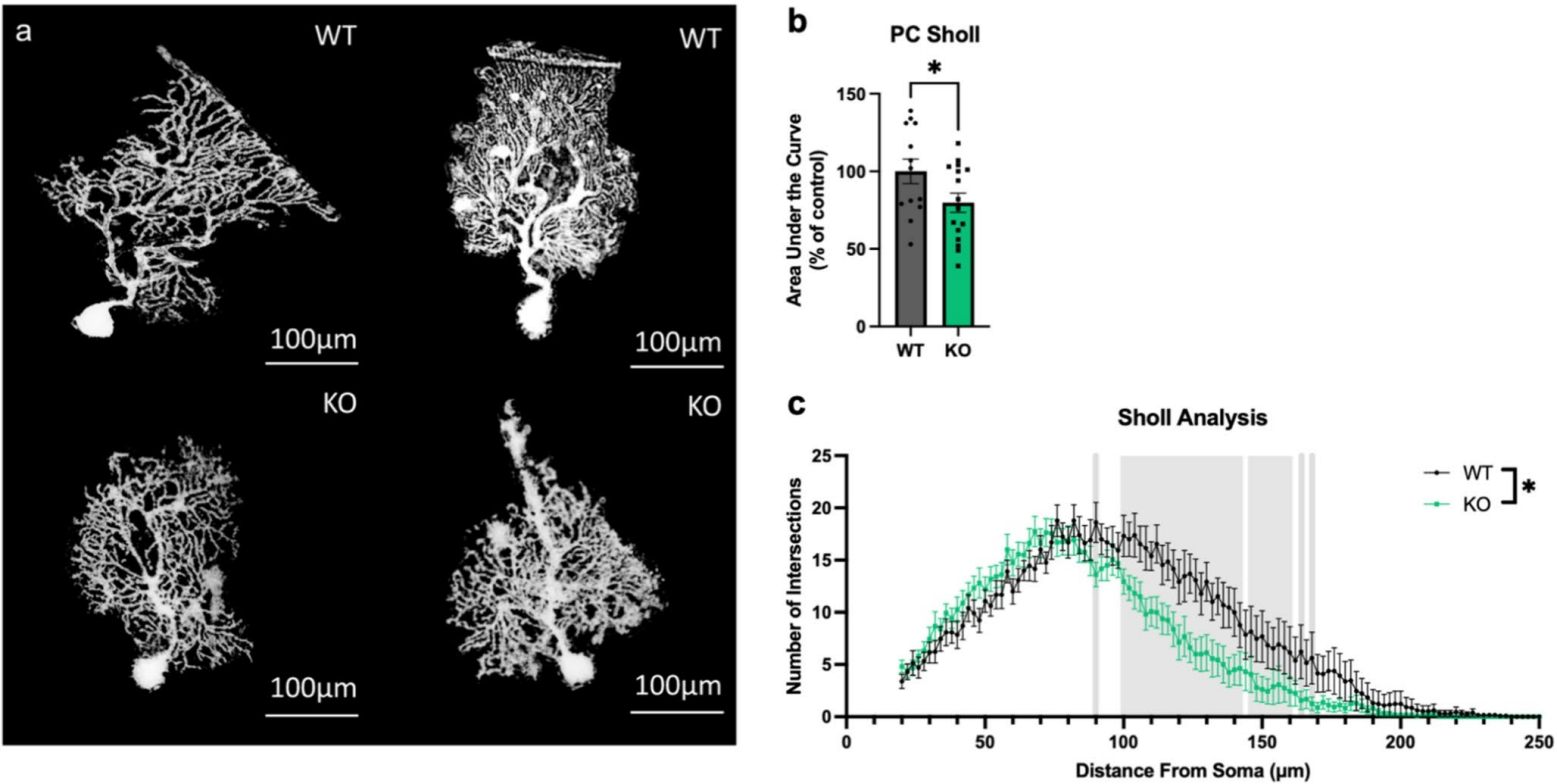
Deletion of Ca_V_1.3 results in less complex distal PC dendritic arborization. (**a**) Golgi staining of PCs from Ca_V_1.3 KO and WT mice from sagittal sections. Deletion of Ca_V_1.3 results in less complex PC dendritic arborization (**b**) driven primarily by differences in the distal dendrites (**c**). Distances with significant differences (*p < 0.05) in number of intersections between Ca_V_1.3 KO and WT are highlighted in grey. Data are expressed as mean ± s.e.m. *N* = 3/genotype, aged 15 weeks

Changes in PC arborization and dendritic spines have been associated with impairments in cerebellar-dependent behaviors [39]. We analyzed PC arborization complexity using Sholl analysis on PCs across the vermis labeled using the Golgi method (Fig. 3a) [18]. We observed that loss of Ca_V_1.3 resulted in fewer intersections when compared to WT, resulting in a significant reduction in the area under the curve when counting all intersections (n_WT_=13PCs/3mice & n_KO_=16PCs/3mice, nested *t* test, main genotype effect, F_1,27_=4.22, *p* < 0.05) (Fig. 3b). To determine where differences appeared in dendritic arbors, we divided the arbors into 2 μm bins (Fig. 3c), which showed that Ca_V_1.3-dependent differences in PC dendritic arborization appear to be driven primarily by the distal arbors (n_WT_=13PCs/3mice & n_KO_=16PCs/3mice, Linear Mixed Effects Model, main genotype effect, F_1,27_=4.15, *p* = 0.05, main radius effect, F_115,3105_=50.83, *p* < 0.01, genotype x radius interaction effect, F_115,3105_=2.77, *p* < 0.01, Estimated Marginal Means, *p* < 0.01 at 90 μm, 100–140 μm, and 150 μm, *p* < 0.05 from 100 to 142 μm, 146–160 μm, 164 μm, and 168 μm).

**Fig. 4:**
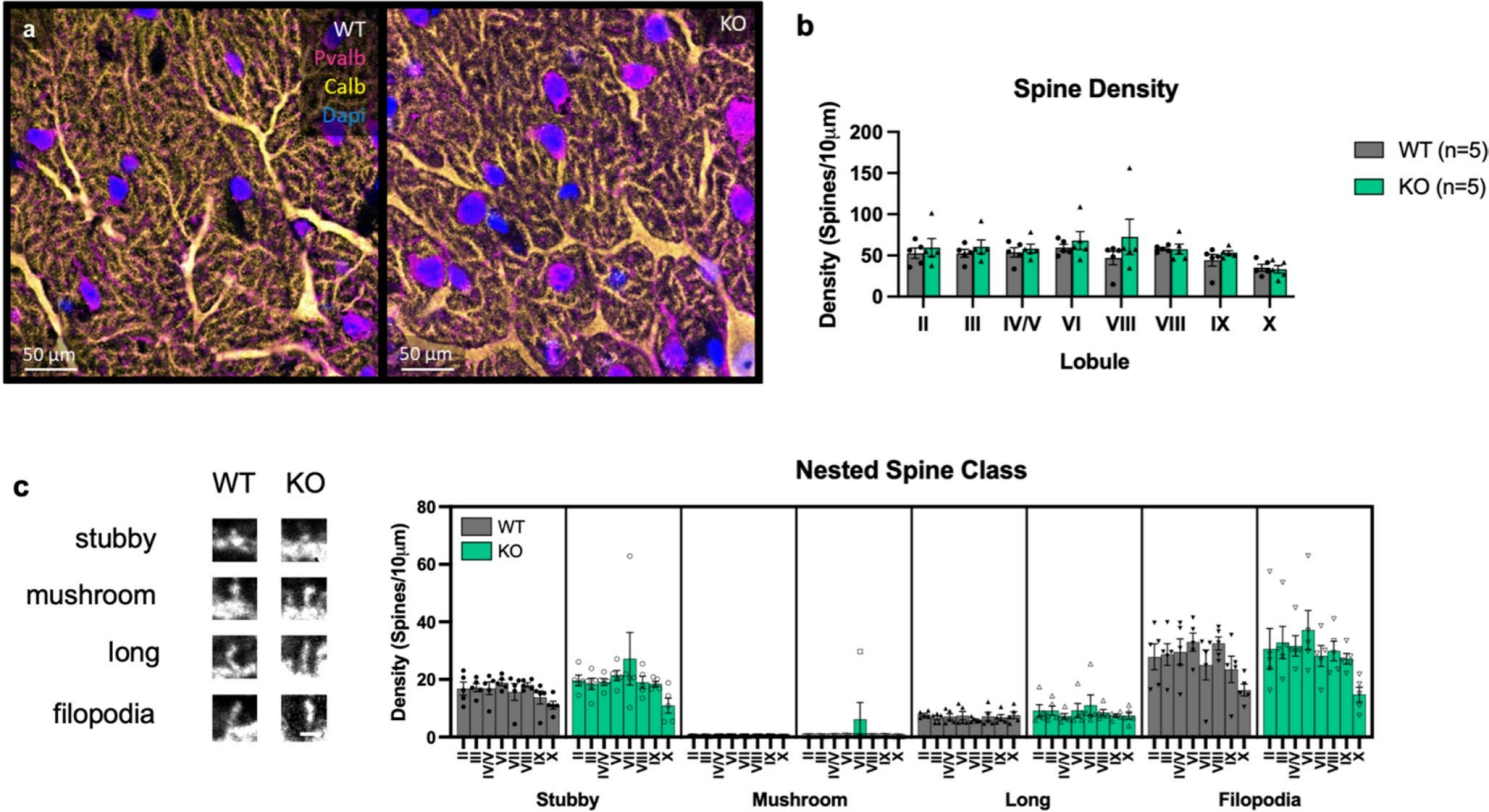
PC spine density varies by lobule. (**a**) Immunofluorescent staining of PV (magenta), calbindin (gold), and DAPI (blue) from Ca_V_1.3 KO and WT mice from sagittal sections. Although spine density varies significantly by lobule, deletion of Ca_V_1.3 does not alter PC spine density (**b**). (**c**) The density of different classes of PC dendritic spines (examples on *left*) does not vary between genotypes. Data are expressed as mean ± s.e.m. *N* = 5/genotype, aged 20–31 weeks

We next sought to determine whether Ca_V_1.3 KO mice display abnormal PC dendritic spines (Fig. 4a). We found that spine density varied by lobule (*n* = 5/genotype, Linear Mixed Effects Model, spine density, main lobule effect, F_7,56_=4.37, *p* < 0.01) (Fig. 4b), as did spine classification (*n* = 5/genotype, Linear Mixed Effects Model, main lobule effect, F_7,248_=7.09, *p* < 0.01, main spine type effect, F_7,248_=389.77, *p* < 0.01, lobule x spine type interaction effect, F_21,248_=2.84, *p* < 0.01) (Fig. 4c). We observed no Ca_V_1.3-dependent differences in PC spine density (*n* = 5/genotype, Linear Mixed Effects Model, main genotype effect, F_1,8_=0.78, *p* = 0.40, genotype x lobule interaction effect, F_7,56_=0.93, *p* = 0.49) (Fig. 4b) nor in distribution of spine classification in any lobule of the vermis (*n* = 5/genotype, Linear Mixed Effects Model, main genotype effect, F_1,8_=0.78, *p* = 0.40, genotype x lobule interaction effect, F_7,248_=1.51, *p* = 0.16, genotype x spine type interaction effect, F_3,248_=0.73, *p* = 0.54, genotype x lobule x spine type interaction effect, F_21,248_=0.28, *p* = 1.00) (Fig. 4c).

### Loss of Ca_V_1.3 does not Alter MLI-PC Synapse Morphology

**Fig. 5:**
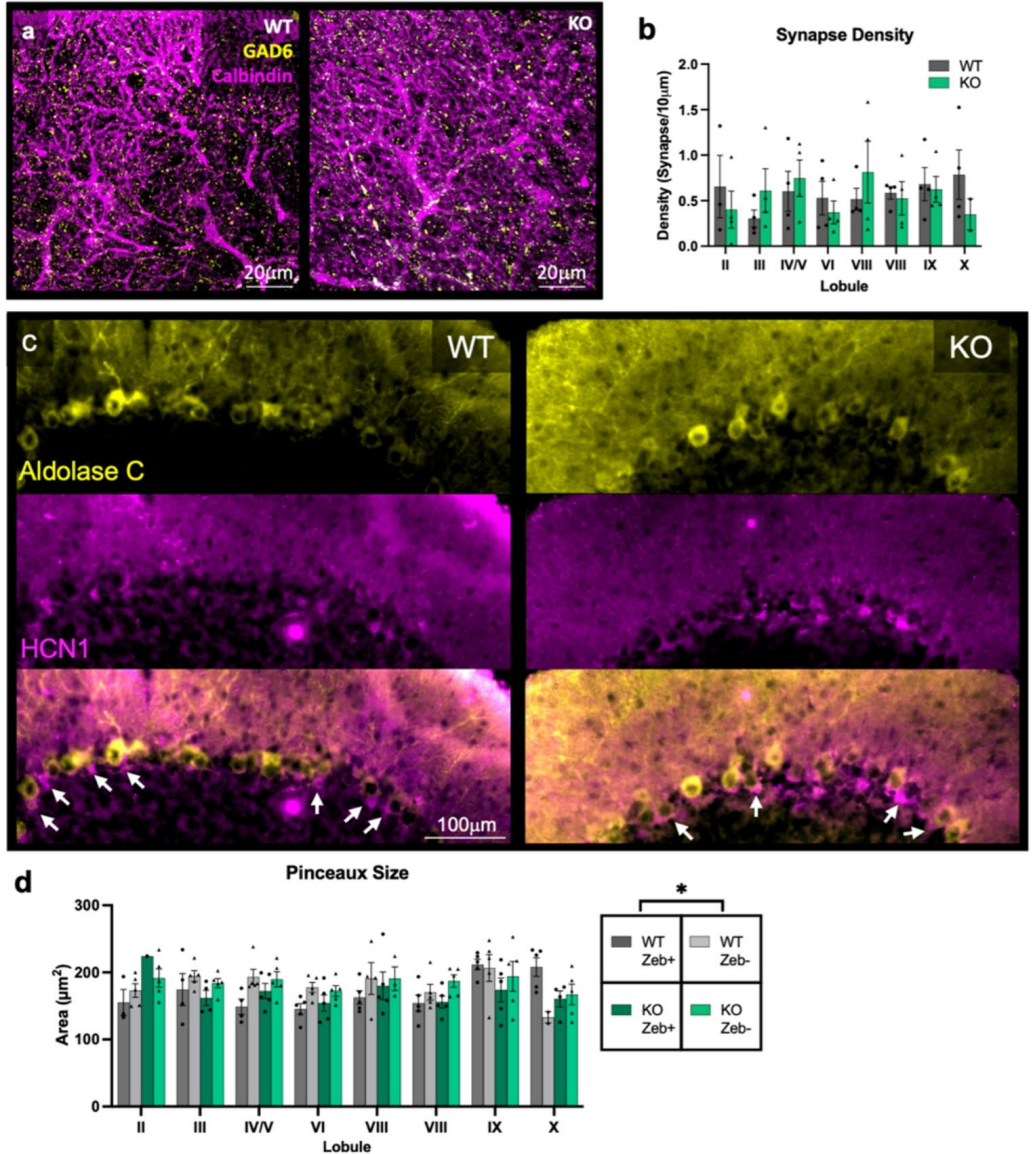
Pinceaux size varies by lobule and zebrin striping. (**a**) Immunofluorescent staining of calbindin (magenta) and GAD6 (gold) from sagittal sections of Ca_V_1.3 KO and WT mice. (**b**) Deletion of Ca_V_1.3 does not alter GABAergic synapse density in the cerebellar molecular layer. *N* = 5/genotype, aged 13–31 weeks. (**c**) Immunofluorescent staining of HCN1 (magenta) and aldolase C (gold) from sagittal sections of Ca_V_1.3 KO and WT mice. White arrows indicate pinceaux. (**d**) Deletion of Ca_V_1.3 does not alter pinceaux size in the cerebellar molecular layer, although pinceaux sizes are generally larger in zebrin-negative zones than zebrin-positive zones. Data are expressed as mean ± s.e.m. *N* = 5/genotype, aged 13–31 weeks

Given the high expression of *Cacna1d* in MLIs, we next sought to determine whether Ca_V_1.3 KO MLIs display abnormal presynaptic structures. We first looked at GABAergic synapse density on PC dendrites as measured by GAD6 puncta on calbindin-positive dendrites (Fig. 5a). We observed no differences in GABAergic synapse density on PC dendrites (*n* = 4/genotype, Linear Mixed Effects Model, main genotype effect, F_1,6_=0.03, *p* = 0.88, main lobule effect, F_7,39_=0.58, *p* = 0.77, genotype x lobule interaction effect, F_7,39_=1.05, *p* = 0.41) (Fig. 5b). GAD6 is not specific to MLI synapses onto PCs, and would also include Golgi cell inputs onto PCs; nevertheless, since no differences were observed, this suggests that presynaptic inputs at GAD6 + synapses are not different between WT and KO mice. We then sought to determine whether Ca_V_1.3 KO mice display abnormal BC pinceau size (Fig. 5c). The size of BC pinceaux is typically distributed into zonal modules and this zonal patterning respects PC zonal boundaries [77]. Specifically, BC pinceaux are smaller in zebrin II-positive PC zones and larger in PLCβ4-positive and NFH-positive PC zones [77]. We replicated this general result (*n* = 5/genotype, Linear Mixed Effects Model, main zebrin effect, F_1,105_=4.05, *p* < 0.05), although this pattern was not observed in lobules IX and X (Fig. 5d). We also observed that BC pinceau size varies by lobule (main lobule effect, F_7,105_=2.97, *p* < 0.01). We observed no main effect of genotype in BC pinceau size between Ca_V_1.3 KO and WT mice controlling for PC zones (*n* = 5/genotype, Linear Mixed Effects Model, main genotype effect, F_1,8_=0.17, *p* = 0.69) although there were some trend level genotype interaction effects that did not meet criteria for statistical significance (genotype x lobule interaction effect, F_7,105_=1.78, *p* = 0.10, genotype x zebrin interaction effect, F_1,105_=0.01, *p* = 0.94, lobule x zebrin interaction effect, F_7,105_=2.05, *p* = 0.06, genotype x lobule x zebrin interaction effect, F_7,105_=1.75, *p* = 0.10) (Fig. 5d).

### Global deletion of Ca_V_1.3 does not Alter Delay Eyeblink Conditioning

**Fig. 6:**
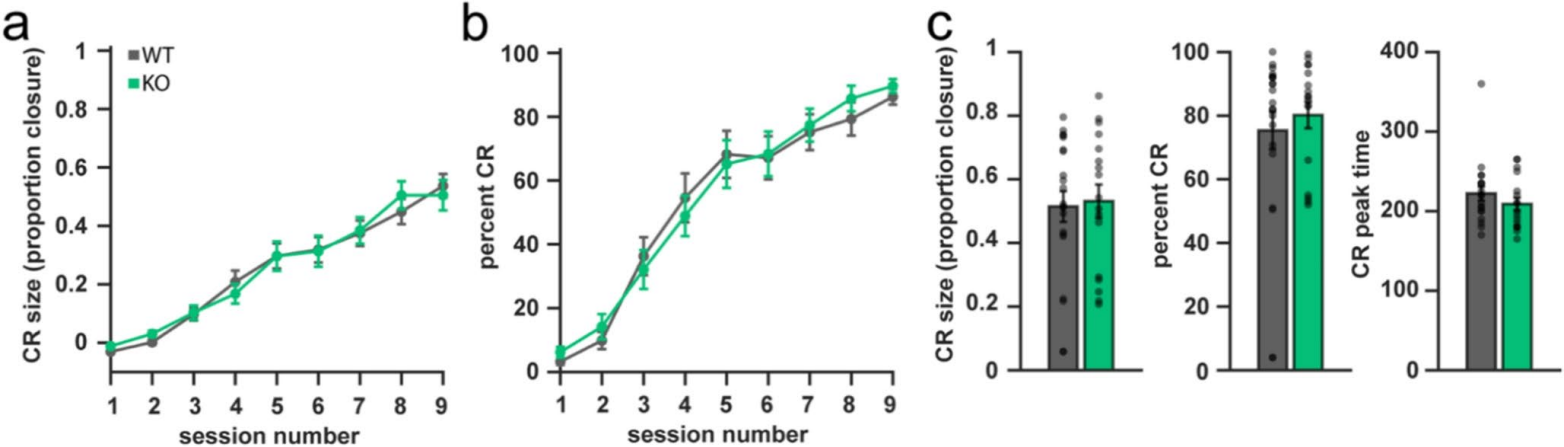
Deletion of Ca_V_1.3 does not impact acquisition of delay eyeblink conditioning. (**a**) No group differences were detected in the learning curves for degree of eye closure during delay eyeblink conditioning training. (**b**) No group differences were detected in the learning curves for conditioned responses during delay eyeblink conditioning training. (**c**) Deletion of Ca_V_1.3 does not alter size of conditioned eyeblink responses (CR size), frequency of conditioned eyeblink responses (percent CR), or timing of conditioned eyeblink responses (CR peak time). Data are expressed as mean ± s.e.m. *N* = 15–17/genotype, aged 20–24 weeks

Given the reduced dendritic complexity in Ca_V_1.3 KO PCs (Fig. 3a), and the importance of PC function in eyeblink conditioning [12, 24, 28], we examined whether deletion of Ca_V_1.3 altered cerebellum-dependent delay eyeblink conditioning. We examined the learning curves for amplitude of eyelid closure, also called the conditioned response (CR), and observed no effects of genotype or sex and no interaction effect (two-way RM ANOVA, genotype effect F_8,152_=0.53, *p* = 0.83, sex effect F_8,152_=1.6, *p* = 0.13, genotype x sex interaction effect, F_8,152_=1.25, *p* = 0.27). We did find a main effect of session which shows that mice learned the task (F_8,152_=75.98, *p* < 0.01) (Fig. 6a). We then looked at the learning curves for CR percentage, another measure of how learning occurred over the course of training. We again observed no differences between WT and Ca_V_1.3 KO mice (two-way RM ANOVA, genotype effect F_8,152_=0.42, *p* = 0.91, sex effect F_8,152_=0.35, *p* = 0.95, genotype x sex interaction effect, F_8,152_=1.45, *p* = 0.18) although there was a main effect of session (F_8,152_=92.64, *p* < 0.01) (Fig. 6b). Looking at just the final day of training, we saw no group differences for CRs in terms of amplitude (two-way ANOVA, main genotype effect, F_1,37_=0.14, *p* = 0.71, main sex effect, F_1,37_=0.0, *p* = 0.98, genotype x sex interaction effect, F_1,37_=0.07, *p* = 0.80), frequency (two-way ANOVA, main genotype effect, F_1,37_=1.72, *p* = 0.20, main sex effect, F_1,37_=1.71, *p* = 0.20, genotype x sex interaction effect, F_1,37_=0.79, *p* = 0.38), or timing (two-way ANOVA, main genotype effect, F_1,35_=1.43, *p* = 0.24, main sex effect, F_1,35_=1.63, *p* = 0.21, genotype x sex interaction effect, F_1,35_=0.02, *p* = 0.89) (Fig. 6c).

## Discussion

Previous work with Ca_V_1.3 KO mice has shown that loss of Ca_V_1.3 results in decreased volume of the auditory brainstem and degeneration of cochlear hair cells without evidence of cerebellar degeneration or atrophy [33]. Consistent with previously published data, our results in general show that loss of Ca_V_1.3 does not alter gross cerebellar structure, particularly with respect to cerebellar cortex thickness and neuronal density. We hypothesized that a loss of Ca_V_1.3 would result in abnormal MLI morphology and alter GABAergic MLI-PC synapses. To our surprise, Ca_V_1.3 deletion alters PC morphology without appearing to affect density of PCs or MLIs, or the synaptic structure of MLI inputs onto PCs.

Ca_V_1.3 is crucial for the survival of several neuronal cell types including cochlear hair cells [33] and hippocampal neurons [38, 45]. It is unknown if Ca_V_1.3 is involved in the survival of cerebellar cortex neurons; however, one study of Ca_V_1.3 KO mice suggests that loss of Ca_V_1.3 does not negatively impact cerebellar cortex surface area in juvenile Ca_V_1.3 KO mice [33]. Our results expand upon this previous study through our careful quantification of PCs and MLIs, finding that Ca_V_1.3 is not essential for normal density of these neuronal types. We also did not observe impacts of Ca_V_1.3 on the cross-sectional thickness of the granule and molecular layers. We did not attempt to specifically quantify other cerebellar cortex cell types, such as Bergmann glia, or other less abundant types of interneurons such as Golgi cells, Lugaro cells, and unipolar brush cells. Of these other cerebellar cortex cell types, only Golgi cells have been reported to have high expression of *Cacna1d* mRNA [41], but we do not know whether there is electrophysiological evidence of L-type activity in Golgi cells. For this reason, we did not prioritize examining them here. In the future, it may be worthwhile to determine whether Golgi cells are impacted by loss of Ca_V_1.3 if evidence of L-type activity in Golgi cells emerges. We also did not examine neurons of the deep cerebellar nuclei, although some of these neurons likely do express Ca_V_1.3. Future studies may also reveal important Ca_V_1.3-dependent differences in these neuronal types.

L-type channels in the brain are most often thought to act primarily postsynaptically, regulating the expression patterns of membrane receptors and the morphology of dendritic spines in an activity-dependent manner [65, 67, 68]. Spines are typically classified into one of four groups: long/thin, stubby, mushroom, and filopodia, each thought to be relevant in different aspects of neurodevelopment and plasticity [52]. For example, mushroom spines are considered the most stable and associated with long-term memory, whereas long/thin spines are thought to be more dynamic and related to active learning. Stubby spines are most common during development or as remnants of mushroom spines when they are eliminated. Filopodia are mobile and flexible, with short lifetimes, and typically do not appear to have mature post-synaptic density machinery under electron microscopy. Notably, this type of classification scheme does not reflect all possible spine shapes, nor do all spines align well with this classification approach [52], but it is a reasonable first approximation regarding whether a specific gene may alter spine shape generally.

Ca_V_1.3 interacts with the PDZ domain of proteins that regulate dendritic spine growth and stability. Hippocampal neurons expressing a mutant Ca_V_1.3 channel lacking this PDZ binding domain display an increase in spine elongation [68]. Loss of Ca_V_1.3 has also been shown to increase dendritic spine complexity and reduce cell body size in auditory brainstem neurons [33]. Our data show that there are no differences in dendritic spines on PCs but there is decreased complexity in the distal dendritic arbors of PCs in Ca_V_1.3 KO mice. It is unclear if this change occurs via a cell-autonomous mechanism (related to intrinsic PC developmental programs) or a cell non-autonomous mechanism (how other cells interact with PCs). Our study did not examine MLI morphology outside of presynaptic structures, so it is possible that changes in MLI size or altered inhibition of PCs by MLIs could lead to the reduced distal PC dendritic complexity we observed in Ca_V_1.3 KO mice. Future studies could differentiate between these two possibilities by utilizing conditional KO lines targeting either PCs or neurons that synapse on PCs such as granule cells, Golgi cells, and MLIs. Our dendritic spine analysis, which we performed using calbindin-labeled PCs, included the entire molecular layer, limiting our ability to determine whether there was any difference in spines depending on the distance from the soma. For analyzing dendritic branching, we sampled Purkinje cells from across the vermis from our Golgi-Cox stained sections, resulting in a random assortment of vermal PCs. It is possible that Purkinje cell dendritic arbors vary as a function of lobule or even position along the folium. Our study was not powered to detect such differences. We also did not measure arborization or dendritic spines of MLIs but given that our results suggest that loss of Ca_V_1.3 alters PC dendritic arborization, Ca_V_1.3 may play a similar role in MLIs or other neuron classes. Future studies may also reveal important Ca_V_1.3-dependent differences in dendrites of other neurons.

In addition to their roles in postsynaptic function, recent work has also found that L-type channels have presynaptic functions in some cell types [17]. For example, in cochlear inner hair cells, Ca_V_1.3 is expressed at the presynaptic ribbon, and influx of Ca^2+^ through Ca_V_1.3 allows for fast and sustained glutamate release [9, 59]. Presynaptic Ca_V_1 channels in hippocampal interneurons and cerebellar MLIs modulate short-term plasticity via regulation of GABA release [36, 60, 68]. Additionally, loss of Ca_V_1.3 alters presynaptic bouton size in the hippocampus [38], suggesting that Ca_V_1.3 has structural as well as electrophysiological presynaptic functions in this structure. However, we did not find any differences in measures of GABAergic MLI-PC synapse number or basket cell pinceau size. These data suggest that Ca_V_1.3 does not regulate presynaptic structure in cerebellar neurons as it does in the hippocampus. In cerebellar slices, when L-type channels are blocked, mIPSCs in MLIs and PCs decrease in frequency; conversely, when L-type channels are activated, mIPSCs in MLIs and PCs increase in frequency [60]. Since Ca_V_1.3 is the major L-type channel expressed in MLIs [41], it is likely the presynaptic L-type channel that modulates cerebellar MLI GABA release.

Taken together these data suggest that Ca_V_1.3 does not alter major aspects of cerebellar anatomy. Instead, our data suggest that Ca_V_1.3 plays an important role in PC structure, which may contribute to some of the behavioral phenotypes previously observed in Ca_V_1.3 KO mice [42]. As noted previously, L-type channel activity has been detected in developing immature PCs [25, 26, 72]. Since our analyses were all performed in adult animals, we cannot say at what stage of development loss of Ca_V_1.3 altered PC structure. Analysis of Ca_V_1.3 KO cerebella at earlier stages of development would be essential for clarifying the origins of the PC dendritic abnormalities in these mice.

It is notable that we do not observe deficits in eyeblink conditioning. We expected loss of PC complexity to impact eyeblink conditioning, so we were surprised to observe normal acquisition, timing, and expression of delay EBC. However, there are examples of the converse, that is, mouse models of autism in which delay EBC is impaired but PC structure appears normal [39]. Global loss of Ca_V_1.3 in mice impairs cognitive function in several systems including development of fear memories and addictive behaviors, working memory and associative memory, and motor learning [7, 10, 35, 38, 42, 45, 46]. Previous work with Ca_V_1.3 KO mice identified impaired consolidation of contextual fear conditioning, though cued fear conditioning was not measured [46] as Ca_V_1.3 KO mice are congenitally deaf [21, 33, 36, 59]. Prior work from our lab found that Ca_V_1.3 KO mice display no changes in motor activity via the open field test but do have impairments in locomotor adaptation and learning as measured by the accelerating rotarod and Erasmus ladder [42]. On the Erasmus ladder, Ca_V_1.3 KO mice display no motor coordination deficits as measured by missteps [42] but do have an impairment in gait adaptation, which is a task linked to cerebellar function [76]. Experiments in PC-specific conditional knockout mice would be required to know how Ca_V_1.3 expression in PCs affects specific behaviors. Interestingly, loss of the non-canonical Wnt signaling protein PRICKLE2 reduces Purkinje cell excitability without affecting eyeblink conditioning [1], suggesting that perhaps larger disturbances of PC activity and structure are required to impact this form of associative learning.

One unanticipated outcome of this work is our findings regarding the variability of cerebellar anatomy across lobules of the vermis. While each lobule contains largely similar neuronal types (n.b. unipolar brush cells which are predominantly observed in lobules IX and X) and cortical layer structure, we found that multiple parameters vary across lobules, including granule and molecular layer thickness, density of MLIs and PCs, and both PC spine density and spine type. Different genetic strains of mice are known to display differences in cerebellar foliation as well [34, 47]. Our data may serve as a reference for further work in this specific genetic background (C57BL6/NTac) and highlight the importance of using comparisons that are matched for genetic strain and lobule in structural analyses.

## Data Availability

The data supporting the findings of this study are available from the corresponding author upon reasonable request.

## References

[1] Abbott PW, Hardie JB, Walsh KP, Nessler AJ, Farley SJ, Freeman JH, et al. Knockdown of the non-canonical Wnt gene Prickle2 leads to cerebellar purkinje cell abnormalities while cerebellar-mediated behaviors remain intact. Cerebellum Lond Engl. 2024. 10.1007/s12311-023-01648-9. 10.1007/s12311-023-01648-9 PMC11217148 38165577

[2] Ament SA, Szelinger S, Glusman G, Ashworth J, Hou L, Akula N, Shekhtman T, Badner JA, Brunkow ME, Mauldin DE, Stittrich A-B, Rouleau K, Detera-Wadleigh SD, Nurnberger JI, Edenberg HJ, Gershon ES, Schork N, Bipolar Genome Study, Price ND, Gelinas R, Hood L, Craig D, McMahon FJ, Kelsoe JR, Roach JC. Rare variants in neuronal excitability genes influence risk for bipolar disorder. Proc Natl Acad Sci U S A. 2015;112:3576–81. 10.1073/pnas.1424958112. 25730879 10.1073/pnas.1424958112 PMC4371952

[3] Andreasen NC. Hypofrontality in schizophrenia: distributed dysfunctional circuits in neuroleptic-naïve patients. Lancet. 1997;349:1730–4. 9193383 10.1016/s0140-6736(96)08258-x

[4] Andreasen NC, Pierson R. The role of the cerebellum in schizophrenia. Biol Psychiatry. 2008;64:81–8. 18395701 10.1016/j.biopsych.2008.01.003 PMC3175494

[5] Baig SM. Loss of Ca(v)1.3 (CACNA1D) function in a human channelopathy with bradycardia and congenital deafness. Nat Neurosci. 2011;14:77–84. 21131953 10.1038/nn.2694

[6] Becker EBE, Stoodley CJ. Autism spectrum disorder and the cerebellum. Int Rev Neurobiol. 2013;113:1–34. 10.1016/B978-0-12-418700-9.00001-0. 24290381 10.1016/B978-0-12-418700-9.00001-0

[7] Berger SM, Bartsch D. The role of L-type voltage-gated calcium channels Cav1.2 and Cav1.3 in normal and pathological brain function. Cell Tissue Res. 2014;357:463–76. 24996399 10.1007/s00441-014-1936-3

[8] Bolbecker AR. Eyeblink conditioning anomalies in bipolar disorder suggest cerebellar dysfunction. Bipolar Disord. 2009;11:19–32. 19133963 10.1111/j.1399-5618.2008.00642.x

[9] Brandt A, Striessnig J, Moser T. CaV1.3 channels are essential for development and presynaptic activity of cochlear inner hair cells. J Neurosci. 2003;23:10832–40. 10.1523/JNEUROSCI.23-34-10832.2003. 14645476 10.1523/JNEUROSCI.23-34-10832.2003 PMC6740966

[10] Busquet P. CaV1.3 L-type Ca2 + channels modulate depression-like behaviour in mice independent of deaf phenotype. Int J Neuropsychopharmacol. 2010;13:499–513. 19664321 10.1017/S1461145709990368

[11] Cajal S. 1911. Histologie Du Système Nerveux De L’homme & Des Vertébrés. Hachette Livre.

[12] Chen AI, Zang K, Masliah E, Reichardt LF. Glutamatergic axon-derived BDNF controls GABAergic synaptic differentiation in the cerebellum. Sci Rep. 2016;6:20201. 10.1038/srep20201. 26830657 10.1038/srep20201 PMC4735332

[13] Crespo-Facorro B, Barbadillo L, Pelayo-Terán JM, Rodríguez-Sánchez JM. Neuropsychological functioning and brain structure in schizophrenia. Int Rev Psychiatry Abingdon Engl. 2007;19:325–36. 10.1080/09540260701486647 17671866

[14] Cross-Disorder Group of the Psychiatric Genomics Consortium. Identification of risk loci with shared effects on five major psychiatric disorders: a genome-wide analysis. Lancet Lond Engl. 2013;381:1371–9. 10.1016/S0140-6736(12)62129-1. 10.1016/S0140-6736(12)62129-1 PMC3714010 23453885

[15] Dacre J, Colligan M, Clarke T, Ammer JJ, Schiemann J, Chamosa-Pino V, Claudi F, Harston JA, Eleftheriou C, Pakan JMP, Huang C-C, Hantman AW, Rochefort NL, Duguid I. A cerebellar-thalamocortical pathway drives behavioral context-dependent movement initiation. Neuron. 2021;109:2326–e23388. 10.1016/j.neuron.2021.05.016. 34146469 10.1016/j.neuron.2021.05.016 PMC8315304

[16] De Zeeuw CI, Berrebi AS. Postsynaptic targets of purkinje cell terminals in the cerebellar and vestibular nuclei of the rat. Eur J Neurosci. 1995;7:2322–33. 10.1111/j.1460-9568.1995.tb00653.x. 8563981 10.1111/j.1460-9568.1995.tb00653.x

[17] Dolphin AC, Lee A. Presynaptic calcium channels: specialized control of synaptic neurotransmitter release. Nat Rev Neurosci. 2020;21:213–29. 32161339 10.1038/s41583-020-0278-2 PMC7873717

[18] Dudink I, White TA, Ardalan M, Mallard C, Ballerin G, Creed SJ, et al. An optimized and detailed step-by-step protocol for the analysis of neuronal morphology in Golgi-stained fetal sheep brain. Dev Neurosci. 2022;44:344–62. 10.1159/000524055. 35447627 10.1159/000524055

[19] Eccles JC, Llinás R, Sasaki K. The excitatory synaptic action of climbing fibres on the purkinje cells of the cerebellum. J Physiol. 1966a;182:268–96. 5944665 10.1113/jphysiol.1966.sp007824 PMC1357472

[20] Eccles JC, Llinás R, Sasaki K. The inhibitory interneurones within the cerebellar cortex. Exp Brain Res. 1966b;1:1–16. 10.1007/BF00235206. 5910941 10.1007/BF00235206

[21] Eckrich S, Hecker D, Sorg K, Blum K, Fischer K, Münkner S, et al. Cochlea-specific deletion of Cav1.3 calcium channels arrests inner hair cell differentiation and unravels pitfalls of conditional mouse models. Front Cell Neurosci. 2019;13:225. 10.3389/fncel.2019.00225. 31178698 10.3389/fncel.2019.00225 PMC6538774

[22] Flanagan SE. A CACNA1D mutation in a patient with persistent hyperinsulinaemic hypoglycaemia, heart defects, and severe hypotonia. Pediatr Diabetes. 2017;18:320–3. 28318089 10.1111/pedi.12512 PMC5434855

[23] Fu JM, Satterstrom FK, Peng M, Brand H, Collins RL, Dong S, Wamsley B, Klei L, Wang L, Hao SP, Stevens CR, Cusick C, Babadi M, Banks E, Collins B, Dodge S, Gabriel SB, Gauthier L, Lee SK, Liang L, Ljungdahl A, Mahjani B, Sloofman L, Smirnov AN, Barbosa M, Betancur C, Brusco A, Chung BHY, Cook EH, Cuccaro ML, Domenici E, Ferrero GB, Gargus JJ, Herman GE, Hertz-Picciotto I, Maciel P, Manoach DS, Passos-Bueno MR, Persico AM, Renieri A, Sutcliffe JS, Tassone F, Trabetti E, Campos G, Cardaropoli S, Carli D, Chan MCY, Fallerini C, Giorgio E, Girardi AC, Hansen-Kiss E, Lee SL, Lintas C, Ludena Y, Nguyen R, Pavinato L, Pericak-Vance M, Pessah IN, Schmidt RJ, Smith M, Costa CIS, Trajkova S, Wang JYT, Yu MHC, Aleksic B, Artomov M, Benetti E, Biscaldi-Schafer M, Børglum AD, Carracedo A, Chiocchetti AG, Coon H, Doan RN, Fernández-Prieto M, Freitag CM, Gerges S, Guter S, Hougaard DM, Hultman CM, Jacob S, Kaartinen M, Kolevzon A, Kushima I, Lehtimäki T, Rizzo CL, Maltman N, Manara M, Meiri G, Menashe I, Miller J, Minshew N, Mosconi M, Ozaki N, Palotie A, Parellada M, Puura K, Reichenberg A, Sandin S, Scherer SW, Schlitt S, Schmitt L, Schneider-Momm K, Siper PM, Suren P, Sweeney JA, Teufel K, Trelles DP, Weiss M, Yuen LA, De Rubeis R, Buxbaum S, Daly JD, Devlin MJ, Roeder K, Sanders SJ, Talkowski ME. Rare coding variation provides insight into the genetic architecture and phenotypic context of autism. Nat Genet. 2022;54:1320–31. 35982160 10.1038/s41588-022-01104-0 PMC9653013

[24] Green JT, Steinmetz JE. Purkinje cell activity in the cerebellar anterior lobe after rabbit eyeblink conditioning. Learn Mem. 2005;12:260–9. 10.1101/lm.89505. 15897252 10.1101/lm.89505 PMC1142454

[25] Gruol DL, Netzeband JG, Quina LA, Blakely-Gonzalez PK. Contribution of L-type channels to Ca2 + regulation of neuronal properties in early developing purkinje neurons. Cerebellum Lond Engl. 2005;4:128–39. 10.1080/14734220510007969. 10.1080/14734220510007969 16035195

[26] Gruol DL, Netzeband JG, Schneeloch J, Gullette CE. L-type Ca2 + channels contribute to current-evoked spike firing and associated Ca2 + signals in cerebellar purkinje neurons. Cerebellum. 2006;5:146–54. 16818389 10.1080/14734220600719692

[27] Guidotti A, Auta J, Davis JM, Di-Giorgi-Gerevini V, Dwivedi Y, Grayson DR, Impagnatiello F, Pandey G, Pesold C, Sharma R, Uzunov D, Costa E. Decrease in reelin and glutamic acid decarboxylase67 (GAD67) expression in schizophrenia and bipolar disorder: a postmortem brain study. Arch Gen Psychiatry. 2000;57:1061–9. 10.1001/archpsyc.57.11.1061. 11074872 10.1001/archpsyc.57.11.1061

[28] Halverson HE, Khilkevich A, Mauk MD. Relating cerebellar purkinje cell activity to the timing and amplitude of conditioned eyelid responses. J Neurosci. 2015;35:7813–32. 10.1523/JNEUROSCI.3663-14.2015. 25995469 10.1523/JNEUROSCI.3663-14.2015 PMC4438128

[29] Häusser M, Clark BA. Tonic synaptic inhibition modulates neuronal output pattern and spatiotemporal synaptic integration. Neuron. 1997;19:665–78. 9331356 10.1016/s0896-6273(00)80379-7

[30] Heiney SA, Kim J, Augustine GJ, Medina JF. Precise control of movement kinematics by optogenetic inhibition of Purkinje cell activity. J Neurosci. 2014;34:2321. 10.1523/JNEUROSCI.4547-13.2014. 24501371 10.1523/JNEUROSCI.4547-13.2014 PMC3913874

[31] Heiney SA, Ohmae S, Kim OA, Medina JF. Single-Unit extracellular recording from the cerebellum during eyeblink conditioning in Head-Fixed mice. In: Sillitoe RV, editor. Extracellular recording approaches, neuromethods. New York, NY: Springer; 2018. pp. 39–71. 10.1007/978-1-4939-7549-5_3. 10.1007/978-1-4939-7549-5_3 PMC6542276 31156292

[32] Hell JW, Westenbroek RE, Warner C, Ahlijanian MK, Prystay W, Gilbert MM, et al. Identification and differential subcellular localization of the neuronal class C and class D L-type calcium channel alpha 1 subunits. J Cell Biol. 1993;123:949–62. 10.1083/jcb.123.4.949. 8227151 10.1083/jcb.123.4.949 PMC2200142

[33] Hirtz JJ, Boesen M, Braun N, Deitmer JW, Kramer F, Lohr C, et al. Cav1.3 calcium channels are required for normal development of the auditory brainstem. J Neurosci. 2011;31:8280–94. 10.1523/JNEUROSCI.5098-10.2011. 21632949 10.1523/JNEUROSCI.5098-10.2011 PMC6622878

[34] Inouye M, Oda SI. Strain-specific variations in the folial pattern of the mouse cerebellum. J Comp Neurol. 1980;190:357–62. 10.1002/cne.901900209. 7381062 10.1002/cne.901900209

[35] Jelitai M, Puggioni P, Ishikawa T, Rinaldi A, Duguid I. Dendritic excitation-inhibition balance shapes cerebellar output during motor behaviour. Nat Commun. 2016;7:13722. 27976716 10.1038/ncomms13722 PMC5172235

[36] Jensen K, Mody I. L-type Ca2 + channel-mediated short-term plasticity of GABAergic synapses. Nat Neurosci. 2001;4:975–6. 11547336 10.1038/nn722

[37] Johnson CP, Christensen GE, Fiedorowicz JG, Mani M, Shaffer JJ, Magnotta VA, et al. Alterations of the cerebellum and basal ganglia in bipolar disorder mood states detected by quantitative T1ρ mapping. Bipolar Disord. 2018;20:381–90. 10.1111/bdi.12581. 29316081 10.1111/bdi.12581 PMC5995598

[38] Kim S-H. Reduction of Cav1.3 channels in dorsal hippocampus impairs the development of dentate gyrus newborn neurons and hippocampal-dependent memory tasks. PLoS ONE. 2017;12:0181138. 10.1371/journal.pone.0181138 PMC5513490 28715454

[39] Kloth AD, Badura A, Li A, Cherskov A, Connolly SG, Giovannucci A, et al. Cerebellar associative sensory learning defects in five mouse autism models. eLife. 2015;4:e06085. 10.7554/eLife.06085. 26158416 10.7554/eLife.06085 PMC4512177

[40] Kozareva V, Martin C, Osorno T, Rudolph S, Guo C, Vanderburg C, Nadaf N, Regev A, Regehr WG, Macosko E. A transcriptomic atlas of mouse cerebellar cortex comprehensively defines cell types. Nature. 2021a;598:214–9. 10.1038/s41586-021-03220-z. 34616064 10.1038/s41586-021-03220-z PMC8494635

[41] Kozareva V, Martin C, Osorno T, Rudolph S, Guo C, Vanderburg C, Nadaf N, Regev A, Regehr WG, Macosko E. A transcriptomic atlas of mouse cerebellar cortex comprehensively defines cell types. Nature. 2021b;598:214–9. 10.1038/s41586-021-03220-z. 34616064 10.1038/s41586-021-03220-z PMC8494635

[42] Lauffer M, Wen H, Myers B, Plumb A, Parker K, Williams A. Deletion of the voltage-gated calcium channel, CaV1.3, causes deficits in motor performance and associative learning. Genes Brain Behav. 2022;21:e12791. 10.1111/gbb.12791. 35044095 10.1111/gbb.12791 PMC9744532

[43] Levitt JJ. Quantitative volumetric MRI study of the cerebellum and vermis in schizophrenia: clinical and cognitive correlates. Am J Psychiatry. 1999;156:1105–7. 10401463 10.1176/ajp.156.7.1105 PMC2845842

[44] Lundin NB, Kim D-J, Tullar RL, Moussa-Tooks AB, Kent JS, Newman SD, et al. Cerebellar activation deficits in schizophrenia during an eyeblink conditioning task. Schizophr Bull Open. 2021. 10.1093/schizbullopen/sgab040. 34541537 10.1093/schizbullopen/sgab040 PMC8443466

[45] Marschallinger J, Sah A, Schmuckermair C, Unger M, Rotheneichner P, Kharitonova M, et al. The l-type calcium channel Cav1.3 is required for proper hippocampal neurogenesis and cognitive functions. Cell Calcium. 2015;58:606–16. 10.1016/j.ceca.2015.09.007. 26459417 10.1016/j.ceca.2015.09.007

[46] McKinney BC, Murphy GG. The L-type voltage-gated calcium channel Cav1.3 mediates consolidation, but not extinction, of contextually conditioned fear in mice. Learn Mem. 2006;13:584–9. 17015855 10.1101/lm.279006 PMC1783612

[47] Neumann PE, Mueller GG, Sidman RL. Identification and mapping of a mouse gene influencing cerebellar folial pattern. Brain Res. 1990;524:85–9. 10.1016/0006-8993(90)90495-w. 2400934 10.1016/0006-8993(90)90495-w

[48] Ortner NJ, Kaserer T, Copeland JN, Striessnig J. De Novo CACNA1D Ca2 + channelopathies: clinical phenotypes and molecular mechanism. Pflugers Arch. 2020;472:755–73. 10.1007/s00424-020-02418-w. 32583268 10.1007/s00424-020-02418-w PMC7351864

[49] Palay S, Chan-Palay V. Cerebellar cortex: cytology and organization. Berlin, Heidelberg, New York: Springer; 1974.

[50] Pardiñas AF, Holmans P, Pocklington AJ, Escott-Price V, Ripke S, Carrera N, Legge SE, Bishop S, Cameron D, Hamshere ML, Han J, Hubbard L, Lynham A, Mantripragada K, Rees E, MacCabe JH, McCarroll SA, Baune BT, Breen G, Byrne EM, Dannlowski U, Eley TC, Hayward C, Martin NG, McIntosh AM, Plomin R, Porteous DJ, Wray NR, Caballero A, Geschwind DH, Huckins LM, Ruderfer DM, Santiago E, Sklar P, Stahl EA, Won H, Agerbo E, Als TD, Andreassen OA, Bækvad-Hansen M, Mortensen PB, Pedersen CB, Børglum AD, Bybjerg-Grauholm J, Djurovic S, Durmishi N, Pedersen MG, Golimbet V, Grove J, Hougaard DM, Mattheisen M, Molden E, Mors O, Nordentoft M, Pejovic-Milovancevic M, Sigurdsson E, Silagadze T, Hansen CS, Stefansson K, Stefansson H, Steinberg S, Tosato S, Werge T, Consortium CRESTARC, Collier DA, Rujescu D, Kirov G, Owen MJ, O’Donovan MC, Walters JTR. Common schizophrenia alleles are enriched in mutation-intolerant genes and in regions under strong background selection. Nat Genet. 2018;50:381–9. 10.1038/s41588-018-0059-2. 29483656 10.1038/s41588-018-0059-2 PMC5918692

[51] Parker KL, Narayanan NS, Andreasen NC. The therapeutic potential of the cerebellum in schizophrenia. Front Syst Neurosci. 2014;15(8):163. 10.3389/fnsys.2014.00163. 10.3389/fnsys.2014.00163 PMC4163988 25309350

[52] Pchitskaya E, Bezprozvanny I. Dendritic spines shape Analysis-Classification or clusterization?? Perspective. Front Synaptic Neurosci. 2020;12:31. 10.3389/fnsyn.2020.00031. 33117142 10.3389/fnsyn.2020.00031 PMC7561369

[53] Perciavalle V, Apps R, Bracha V, Delgado-García JM, Gibson AR, Leggio M, Carrel AJ, Cerminara N, Coco M, Gruart A, Sánchez-Campusano R. Consensus paper: current views on the role of cerebellar interpositus nucleus in movement control and emotion. Cerebellum Lond Engl. 2013;12:738–57. 10.1007/s12311-013-0464-0. 10.1007/s12311-013-0464-0 23564049

[54] Pinggera A. CACNA1D de novo mutations in autism spectrum disorders activate Cav1.3 L-type calcium channels. Biol Psychiatry. 2015;77:816–22. 25620733 10.1016/j.biopsych.2014.11.020 PMC4401440

[55] Pinggera A. New gain-of-function mutation shows CACNA1D as recurrently mutated gene in autism spectrum disorders and epilepsy. Hum Mol Genet. 2017;26:2923–32. 28472301 10.1093/hmg/ddx175 PMC5886262

[56] Pinggera A. Gating defects of disease-causing de novo mutations in Cav1.3 Ca2 + channels. Channels Austin Tex. 2018;12:388–402. 30465465 10.1080/19336950.2018.1546518 PMC6287693

[57] Pinggera A, Striessnig. L-type Ca2 + channel dysfunction in CNS disorders. J Cav. 2016;1:5839–49. 10.1113/JP270672 PMC4823145 26842699

[58] Pinggera A, Lieb A, Benedetti B, Lampert M, Monteleone S, Liedl KR, et al. CACNA1D de novo mutations in autism spectrum disorders activate Cav1.3 L-type calcium channels. Biol Psychiatry. 2015;77:816–22. 10.1016/j.biopsych.2014.11.020. 25620733 10.1016/j.biopsych.2014.11.020 PMC4401440

[59] Platzer J, Engel J, Schrott-Fischer A, Stephan K, Bova S, Chen H, Zheng H, Striessnig J. Congenital deafness and sinoatrial node dysfunction in mice lacking class D L-type Ca2 + channels. Cell. 2000;102:89–97. 10.1016/s0092-8674(00)00013-1. 10929716 10.1016/s0092-8674(00)00013-1

[60] Rey S, Maton G, Satake S, Llano I, Kang S, Surmeier DJ, Silverman RB, Collin T. Physiological involvement of presynaptic L-type voltage‐dependent calcium channels in GABA release of cerebellar molecular layer interneurons. J Neurochem. 2020;155:390–402. 10.1111/jnc.15100. 32491217 10.1111/jnc.15100

[61] Ross J, Gedvilaite E, Badner JA, Erdman C, Baird L, Matsunami N, et al. A rare variant in CACNA1D segregates with 7 bipolar I disorder cases in a large pedigree. Complex Psychiatry. 2016;2:145–50. 10.1159/000448041. 10.1159/000448041 PMC5109989 27867939

[62] Saunders A, Macosko EZ, Wysoker A, Goldman M, Krienen FM, de Rivera H, Bien E, Baum M, Bortolin L, Wang S, Goeva A, Nemesh J, Kamitaki N, Brumbaugh S, Kulp D, McCarroll SA. Molecular diversity and specializations among the cells of the adult mouse brain. Cell. 2018;174:1015–e103016. 10.1016/j.cell.2018.07.028. 30096299 10.1016/j.cell.2018.07.028 PMC6447408

[63] Schmahmann JD, Sherman JC. The cerebellar cognitive affective syndrome. Brain J Neurol. 1998;121(Pt 4):561–79. 10.1093/brain/121.4.561. 10.1093/brain/121.4.561 9577385

[64] Scholl UI. Somatic and germline CACNA1D calcium channel mutations in aldosterone-producing adenomas and primary aldosteronism. Nat Genet. 2013;45:1050–4. 23913001 10.1038/ng.2695 PMC3876926

[65] Shah MM, Hammond RS, Hoffman DA. Dendritic ion channel trafficking and plasticity. Trends Neurosci. 2010;33:307–16. 20363038 10.1016/j.tins.2010.03.002 PMC2902701

[66] Shinn AK. Aberrant cerebellar connectivity in bipolar disorder with psychosis. Biological Psychiatry: Cognitive Neuroscience and Neuroimaging. 2017;2:438–48. 28730183 10.1016/j.bpsc.2016.07.002 PMC5512437

[67] Stanika RI, Flucher BE, Obermair GJ. Regulation of postsynaptic stability by the L-type calcium channel CaV1.3 and its interaction with PDZ proteins. Curr Mol Pharmacol. 2015;8:95–101. 10.2174/1874467208666150507103716. 25966696 10.2174/1874467208666150507103716 PMC5384370

[68] Stanika R, Campiglio M, Pinggera A, Lee A, Striessnig J, Flucher BE, Obermair GJ. Splice variants of the CaV1.3 L-type calcium channel regulate dendritic spine morphology. Sci Rep. 2016;6:34528. 10.1038/srep34528. 27708393 10.1038/srep34528 PMC5052568

[69] Stoodley CJ, D’Mello AM, Ellegood J, Jakkamsetti V, Liu P, Nebel MB, Gibson JM, Kelly E, Meng F, Cano CA, Pascual JM, Mostofsky SH, Lerch JP, Tsai PT. Altered cerebellar connectivity in autism and cerebellar-mediated rescue of autism-related behaviors in mice. Nat Neurosci. 2017;20:1744–51. 10.1038/s41593-017-0004-1. 29184200 10.1038/s41593-017-0004-1 PMC5867894

[70] Tan GC. Aldosterone-producing adenomas: histopathology-genotype correlation and identification of a novel CACNA1D mutation. Hypertension. 2017;70:129–36. 28584016 10.1161/HYPERTENSIONAHA.117.09057

[71] Tedesco AM, Chiricozzi FR, Clausi S, Lupo M, Molinari M, Leggio MG. The cerebellar cognitive profile. Brain. 2011;134:3672–86. 10.1093/brain/awr266. 22036960 10.1093/brain/awr266

[72] Tringham EW, Payne CE, Dupere JRB, Usowicz MM. Maturation of rat cerebellar purkinje cells reveals an atypical Ca2 + channel current that is inhibited by omega-agatoxin IVA and the dihydropyridine (-)-(S)-Bay K8644. J Physiol. 2007;578:693–714. 17124267 10.1113/jphysiol.2006.121905 PMC2151333

[73] Turner BM, Paradiso S, Marvel CL, Pierson R, Boles Ponto LL, Hichwa RD, Robinson RG. The cerebellum and emotional experience. Neuropsychologia. 2007;45:1331–41. 10.1016/j.neuropsychologia.2006.09.023. 17123557 10.1016/j.neuropsychologia.2006.09.023 PMC1868674

[74] Uhlén M, Fagerberg L, Hallström BM, Lindskog C, Oksvold P, Mardinoglu A, Sivertsson Å, Kampf C, Sjöstedt E, Asplund A, Olsson I, Edlund K, Lundberg E, Navani S, Szigyarto CA-K, Odeberg J, Djureinovic D, Takanen JO, Hober S, Alm T, Edqvist P-H, Berling H, Tegel H, Mulder J, Rockberg J, Nilsson P, Schwenk JM, Hamsten M, von Feilitzen K, Forsberg M, Persson L, Johansson F, Zwahlen M, von Heijne G, Nielsen J, Pontén F. Proteomics. Tissue-based map of the human proteome. Science. 2015;347:1260419. 10.1126/science.1260419. 25613900 10.1126/science.1260419

[75] Uusisaari M, De Schutter E. The mysterious microcircuitry of the cerebellar nuclei. J Physiol. 2011;589:3441–57. 10.1113/jphysiol.2010.201582. 21521761 10.1113/jphysiol.2010.201582 PMC3167109

[76] Vinueza Veloz MF, Zhou K, Bosman LWJ, Potters J-W, Negrello M, Seepers RM, Strydis C, Koekkoek SKE, De Zeeuw CI. Cerebellar control of gait and interlimb coordination. Brain Struct Funct. 2015;220:3513–36. 10.1007/s00429-014-0870-1. 25139623 10.1007/s00429-014-0870-1 PMC4575700

[77] Zhou J, Brown AM, Lackey EP, Arancillo M, Lin T, Sillitoe RV. Purkinje cell neurotransmission patterns cerebellar basket cells into zonal modules defined by distinct pinceau sizes. eLife. 2020;9:e55569. 10.7554/eLife.55569. 32990595 10.7554/eLife.55569 PMC7561353

